# Overexpression of *UHRF1* promotes silencing of tumor suppressor genes and predicts outcome in hepatoblastoma

**DOI:** 10.1186/s13148-018-0462-7

**Published:** 2018-03-02

**Authors:** Alexander Beck, Franziska Trippel, Alexandra Wagner, Saskia Joppien, Max Felle, Christian Vokuhl, Thomas Schwarzmayr, Tim M. Strom, Dietrich von Schweinitz, Gernot Längst, Roland Kappler

**Affiliations:** 1 Department of Pediatric Surgery, Dr. von Hauner Children’s Hospital, Ludwig-Maximilians-University Munich, Lindwurmstr. 2a, 80337 Munich, Germany; 2 Department of Biochemistry III, University Regensburg, Regensburg, Germany; 3 Institute of Paidopathology, Pediatric Tumor Registry, Christian-Albrecht’s-University Kiel, Kiel, Germany; 4 Institute of Human Genetics, Helmholtz Zentrum München, Neuherberg, Germany; 5 Institute of Human Genetics, Technische Universität München, Munich, Germany

**Keywords:** UHRF1, Hepatoblastoma, DNMT1, USP7, Histone methylation, DNA methylation, Epigenetic silencing, Tumor suppressor genes, Biomarker

## Abstract

**Background:**

Hepatoblastoma (HB) is the most common liver tumor of childhood and occurs predominantly within the first 3 years of life. In accordance to its early manifestation, HB has been described to display an extremely low mutation rate. As substitute, epigenetic modifiers seem to play an exceptional role in its tumorigenesis, which holds promise to develop targeted therapies and establish biomarkers for patient risk stratification.

**Results:**

We examined the role of a newly described protein complex consisting of three epigenetic regulators, namely E3 ubiquitin-like containing PHD and RING finger domain 1 (UHRF1), ubiquitin-specific-processing protease 7 (USP7), and DNA methyltransferase 1 (DNMT1), in HB. We found the complex to be located on the promoter regions of the pivotal HB-associated tumor suppressor genes (TSGs) *HHIP*, *IGFBP3*, and *SFRP1* in HB cells, thereby leading to strong repression through DNA methylation and histone modifications. Consequently, knockdown of *UHRF1* led to DNA demethylation and loss of the repressive H3K9me2 histone mark at the TSG loci with their subsequent transcriptional reactivation. The observed growth impairment of HB cells upon *UHRF1* knockdown could be attributed to reduced expression of genes involved in cell cycle progression, negative regulation of cell death, LIN28B signaling, and the adverse 16-gene signature, as revealed by global RNA sequencing. Clinically, overexpression of *UHRF1* in primary tumor tissues was significantly associated with poor survival and the prognostic high-risk 16-gene signature.

**Conclusion:**

These findings suggest that UHRF1 is critical for aberrant TSG silencing and sustained growth signaling in HB and that *UHRF1* overexpression levels might serve as a prognostic biomarker and potential molecular target for HB patients.

**Electronic supplementary material:**

The online version of this article (10.1186/s13148-018-0462-7) contains supplementary material, which is available to authorized users.

## Background

Hepatoblastoma (HB) is the most common liver tumor of childhood, with the majority of cases occurring in children under the age of 3 years [1]. Over the last four decades, advances in treatment protocols comprising chemotherapy and surgery drastically improved outcome of HB patients from a 30% to a roughly 80% overall survival rate [2]. However, there is still a relevant subgroup of high-risk patients presenting with distant metastasis, vascular invasion, advanced tumor stages, or unfavorable histology, whose outcome remains poor [3, 4]. In recent years, molecular markers have been shown to improve HB risk stratification and the effort to find robust markers that can predict outcome in HB patients continues [5, 6].

Molecularly, the development of this aggressive embryonal tumor remains largely unknown. Exome sequencing of primary HB revealed a surprisingly low mutation rate of only 2.9 mutations per tumor on average, and there is evidence that epigenetic dysregulation plays a key role in HB development and progression [7]. Aberrant epigenetic silencing of tumor suppressor genes (TSGs) and overexpression of epigenetic regulators of gene silencing such as histone deacetylases (HDACs) appear to be important drivers of this disease [8]. In particular, the silencing of the TSGs hedgehog-interacting protein (*HHIP*), insulin-like growth factor-binding protein 3 (*IGFBP3*), and secreted frizzled-related protein 1 (*SFRP1*) have been shown to deregulate pivotal pathways of embryonic development, thus promoting HB pathogenesis [9, 10]. While there is evidence that DNA methylation and repressive histone modifications are responsible for aberrant silencing of those genes, the molecular mechanisms through which those epigenetic changes are conveyed and maintained remain largely elusive.

**Fig. 1:**
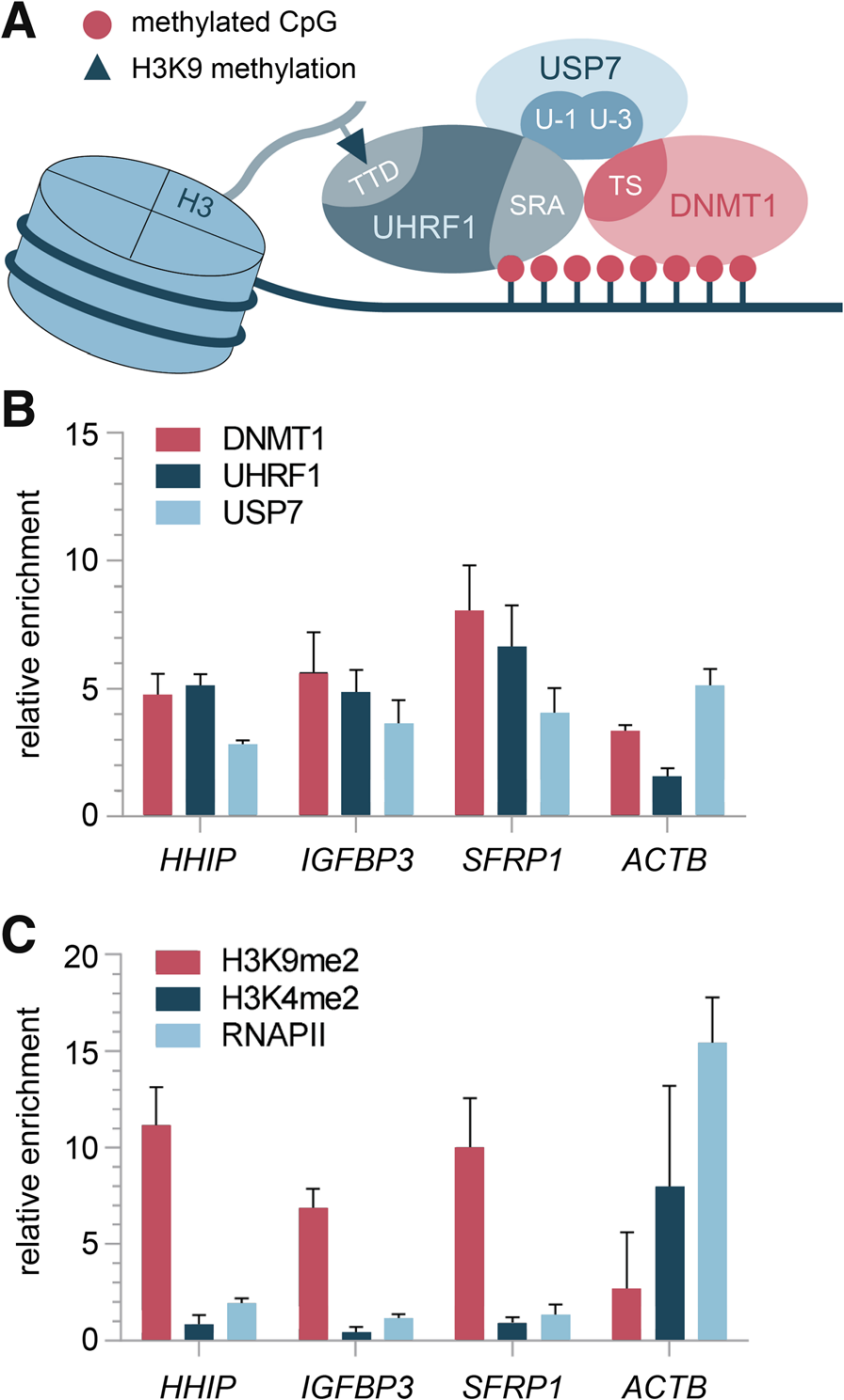
**a** Schematic overview of the trimeric complex comprising UHRF1, USP7, and DNMT1. Involved protein interactions and protein domains according to Felle et al. are shown [11]. **b**, **c** Chromatin immunoprecipitation (ChIP) was performed with HUH6 cells and the indicated antibodies. The average of two independent ChIP experiments for **b** DNMT1, UHRF1, and USP7 as well as **c** H3K9me2, H3K4me2, and RNAPII is shown. Standard deviations, genes of interest, and antibodies used for ChIP are indicated. The enrichment of specific IP versus IgG background is plotted

We recently discovered a protein complex that is involved in aberrant DNA methylation and repression of TSGs in a human colon adenocarcinoma cell line [11]. The complex comprises three subunits, namely DNA methyltransferase 1 (DNMT1), ubiquitin-specific-processing protease 7 (USP7), and E3 ubiquitin-like containing PHD and RING finger domain 1 (UHRF1) (Fig. 1a). Those three proteins are capable of forming a trimeric repression complex through their specific binding domains and carry out distinct functions within the complex. DNMT1 effectively methylates DNA on CpG islands and maintains DNA methylation patterns throughout the cell cycle [12, 13]. USP7 has been shown to enhance DNMT1 function and to stabilize UHRF1 levels by preventing its degradation [11]. While UHRF1 has no chromatin or DNA methylation activities itself, it functions as a crucial guide for DNMT1 and USP7 by binding to both via its SET- and RING-associated (SRA) domain. Through this domain, UHRF1 is also capable of recognizing hemi-methylated DNA, thereby recruiting the complex to hemi-methylated CpGs and maintaining DNA methylation throughout the genome [14].

Furthermore, UHRF1 is involved in histone modification, as it recognizes the repressive histone H3 lysine 9 methylation mark (H3K9me2) via its tandem tudor domain (TTD), thus facilitating the maintenance of this mark and the formation of heterochromatin [15, 16]. UHRF1 is also able to form a complex with HDAC1, another facilitator of histone-driven gene repression [17]. Because of this dynamic crosstalk between histone and DNA modifications, UHRF1 occupies a unique role when it comes to long-term silencing of genes [18].

Overexpression and dysregulation of UHRF1 are common features in a variety of human malignancies, and UHRF1-mediated silencing of TSGs appears to drive tumorigenesis in a disease-specific context [19]. In fact, UHRF1 is so widely overexpressed in solid tumors that it has been suggested as a universal biomarker for cancer [20]. However, the challenge lies in deciphering the disease-specific context in which UHRF1 conveys its oncogenic properties.

Here, we show the trimeric UHRF1 complex to be located on the promoter regions of *HHIP*, *IGFBP3*, and *SFRP1* in HB cells, thereby leading to strong repression of those pivotal HB-associated TSGs through DNA methylation and histone modifications. Knockdown of *UHRF1* results in demethylation of promoter regions, loss of the repressive H3K9me2 mark, and subsequent re-expression of the abovementioned TSGs in HB cells. Furthermore, we show that *UHRF1* knockdown facilitates proliferation impairment and global transcriptional changes in HB cells. Finally, we reveal overexpression of *UHRF1* in primary HB to be associated with poor patient outcome and established risk stratification criteria. These findings suggest that UHRF1 is critical for aberrant TSG silencing and sustained growth signaling in HB and that *UHRF1* expression levels might serve as a prognostic biomarker for HB patients.

## Methods

### Patients

A total of 40 liver tumor specimens were obtained from pediatric patients undergoing surgical resection in our department. Matching normal liver was available from seven patients. Written informed consent was obtained from each patient, and the study protocol was approved by the Committee of Ethics of the Ludwig Maximilian University of Munich.

### Cell culture and transfection

The human hepatoblastoma cell lines HUH6 (Japanese Collection of Research Bioresources, JCRB, Osaka, Japan), HepT1 (provided by Dr. T. Pietsch), and HepG2 (ATCC, Manassas, USA) were cultured under standard conditions in RPMI 1640 growth media (Invitrogen, Carlsbad, CA, USA), supplemented with 10% fetal calf serum, 100 U/mL penicillin, and 100 μg/mL streptomycin at 37 °C in a 5% CO_2_ incubator. For knockdown experiments, 2 × 10^6^ cells were electroporated for 10 ms at 350 V with either siRNA targeting *UHRF1* (siRNA #s26553, Applied Biosystems, Darmstadt, Germany) or appropriate negative control siRNA (siGENOMEN non targeting siRNA #1, Thermo Scientific, Karlsruhe, Germany) in a final concentration of 80 pmol.

### RNA isolation, reverse transcription, and qRT-PCR

Total RNA was isolated 48 h after transfection using TriReagent (Sigma-Aldrich, Steinheim, Germany) according to the manufacturer’s instructions. Two micrograms of total RNA was reverse transcribed into cDNA using random hexamer primer (Roche Diagnostics, Penzberg, Germany) and SuperScriptII Reverse Transcriptase (Invitrogen, Karlsruhe, Germany). PCR amplifications on the Master cycler ep gradient (Eppendorf, Hamburg, Germany) using iTaq-SYBR Green-Supermix (Bio-Rad, Hercules, CA) were performed in doublets as described previously [21]. The following primer pairs (5′–> 3′ orientation) were used: UHRF1, CTCCACGTCCAGGCCG, TGGAGTTCATCTGGACCACG; HHIP, TGTACATCATTCTTGGTGATGGG, AGCCGTAGCACTGAGCCTGT; IGFBP3, GTCCAAGCGGGAGACAGAATAT, CCTGGGACTCAGCACATTGA; SFRP1, CATGACGCCGCCCAAT, GATGGCCTCAGATTTCAACTCG; and TBP, GCCCGAAACGCCGAATAT, CCGTGGTTCGTGGCTCTCT. Data were normalized to the expression level of the housekeeping gene TATA-Box-binding protein (TBP). For calculation of the relative mRNA expression level, the ∆∆CT method was used and expressed as fold change relative to the corresponding control sample [22].

### Western blot analysis

Forty-eight hours after transfection, cells were harvested with ice-cold lysis buffer (0.2 M KCl, 0.03 M Tris, pH 7.25) containing proteinase inhibitor cocktail (Roche Diagnostics). Lysates were boiled in equal volumes of loading buffer (125 mM Tris-HCl, pH 6.8, 4% SDS, 20% glycerol, and 10% ß-mercaptoethanol), and 20 μg of protein was separated in a 8–12% gradient Tris-glycine gradient gel (Novex, San Diego, CA, USA) under reducing conditions and transferred to nitrocellulose membranes (GE Healthcare, Piscataway, USA). Afterwards, membranes were blocked at room temperature with PBS containing 0.1% Tween and 5% non-fat dry milk to block non-specific binding for 2 h. Membranes were incubated with primary antibodies (rabbit anti-human ß-actin, Cell Signaling Technology, Danvers, USA; mouse anti-human UHRF1, generously provided by Dr. C. Bronner, Institute of Genetics and Molecular and Cellular Biology, Strasbourg, France) at 4 °C overnight, followed by a 1-h incubation with the corresponding secondary antibodies (goat anti-rabbit-HRP and goat anti-mouse-HRP, both Dako Cytomation, Hamburg, Germany) at room temperature. For chemiluminescent detection, the ECL Plus Western detection kit (GE Healthcare) was used. Protein bands were detected by autoradiography using the high-performance autoradiography Hyperfilm™ MP (GE Healthcare).

### Methylation-specific polymerase chain reaction (MSP)

Genomic DNA was extracted by phenol/chloroform after proteinase K treatment. Two micrograms of purified genomic DNA was used for bisulfite-mediated conversion of unmethylated cytosine using the Epitec Bisulfite Kit (Qiagen, Hilden, Germany). For MSP, bisulfite-treated DNA was amplified with primers specific for the methylated (M) and unmethylated (U) promoter region of either the *HHIP* (from − 230 to − 87 bp upstream of the transcriptional start site), the *IGFBP3* (from − 180 to − 13 bp upstream of the transcriptional start site), or the *SFRP1* (from + 36 to + 175 bp downstream of the transcriptional start site) gene. As a control for MSP, we used in vitro methylated genomic DNA that has been treated for 4 h with 40 U CpG methyltransferase (M. SssI), NEBuffer2 (10×), and SAM (1:20) at 37 °C, followed by heat inactivation for 20 min at 65 °C. For MSP, we used 1 U hot start Taq polymerase (Thermo Scientific, Schwerte, Germany), 1× hot start Taq buffer, 2 mM dNTPs, 1.5 mM MgCl_2_, 100 ng bisulfite-treated DNA, and 500 nM of the following forward and reverse primers (5′–> 3′ orientation) and annealing temperatures: HHIP-M-F, AGTAGTCGGGTATGTTCGGAATTTTC and HHIP-M-R, GAACCTTCGAAACCAACCTCG at 53 °C; IGFBP3-M-F, GCGAGTTTCGAGTTGTACGTTTTC and IGFBP3-M-R, GCCGACCGCTATATAAAAACCG at 61 °C; SFRP1-M-F, TTTGTAGTTTTCGGAGTTAGTGTCGC and SFRP1-M-R, CGACCCTCGACCTACGATCG at 58 °C; HHIP-U-F, TTGTAGTAGTTGGGTAGTTTTGGAATTTTT and HHIP-U-R, AAACCTTAAAACCAACCTCAAAA at 53 °C; IGFBP3-U-F, TTGGGTGAGTTTTGAGTTGTATGTTTTT and IGFBP3-U-R, AAACACACCAACCACTATATAAAAACCAAA at 61 °C; and SFRP1-U-F, TTTTGTAGTTTTTGGAGTTAGTGTTGTGTG and SFRP1-U-R, CAATAACAACCCTCAACCTACAATCAA at 58 °C. MSP primer design was accomplished using Methyl Primer Express (Applied Biosystems) using the following criteria: CpG percentage > 55%, observed/expected CpG > 65%, and CpG length > 300 bp. MSP reactions were carried out at the following conditions: hot start at 94 °C for 4 min, followed by 38 cycles of 94 °C for 30 s, gene-specific annealing temperature (see above) for 30 s, 72 °C for 45 s, and final extension at 72 °C for 10 min. One percent agarose gel electrophoresis was performed to visualize DNA amplicons.

### Pyrosequencing

For pyrosequencing, 100 ng bisulfite-treated DNA was first amplified in a PCR reaction, using 1 U hot start Taq polymerase (Thermo Scientific), 1× hot start Taq buffer, 2 mM dNTPs, 1.5 mM MgCl_2_, 100 ng bisulfite-treated DNA, and 500 nM of the following forward and reverse primers (5′–> 3′ orientation) and annealing temperatures: HHIP-F, GGGAGGAGAGAGGAGTTT and HHIP-R, AACCAACCTCCAAAATACTAAACC at 55 °C; IGFBP3-F, TGGTTTTTTGAGATTTAAATGTAAGTTAGA and IGFBP3-R, ATCACCCCAATCACTCCTA at 57 °C; SFRP1-F, GGAGTTAGAGATTAGTTTGGTTAATATGG and SFRP1-R, AAAAACCTAAATCATACTTACAACC at 54 °C; and LINE1-F and LINE1-R (assay X58075, Qiagen) at 55 °C. PCR reactions were run at 95 °C for 4 min and 45 cycles of 95 °C for 20 s, gene-specific annealing temperature (see above) for 20 s, and 72 °C for 30 s. PCR products were sequenced with the corresponding sequencing primers HHIP-Seq, TTTAGGATTGAGTTTTTGTTTTAAG; IGFBP3-Seq, TTGGGTTATTTAGGTTTTATATAG; and SFRP1-Seq, GGTAAGAGGTTGTAATTTTAGTTAT using PyroMark Gold Q24 reagents (Qiagen).

### Chromatin immunoprecipitation (ChIP)

8.0 × 10^6^ HUH6 cells were transfected with siRNA against *UHRF1* or corresponding non-target siRNA and subsequently seeded in 15-cm plates. After 48 h, the protein-DNA complexes were cross-linked with 1% formaldehyde for 10 min. The cross-linked reaction was quenched with Glycine Stop-Fix Solution for 5 min. Cell lysis, enzymatic digest (5 min), chromatin immunoprecipitation with 2 μg of antibody against di-methylated H3K4, di-methylated H3K9, RNA-Pol2 (all from Active Motif, La Hulpe, Belgium), and mouse IgG (Santa Cruz, Heidelberg, Germany), respectively, as well as final elution, cross-link reversal, and proteinase K digestion, were performed according to the manufacturer’s protocol (catalog no. 53009, Active Motif). Chromatin samples were subjected to a DNA clean-up step using the QIAquick PCR Purification kit (QIAGEN). Real-time PCR was performed on purified DNA from each of the ChIP reactions using primer pairs for loci of the promoter region of the *HHIP* gene, the *IGFBP3* gene, the *SFRP1* gene, and the *ACTB* gene. Primer pairs used in this study were the following: HHIP_ChIP_F, TTCCCACCTCCTACGGCC and HHIP_ChIP_R, TCCTCTCTCCTCCCCGCTT; IGFBP3_ChIP_F, GCTCCCTGAGACCCAAATGTAA and IGFBP3_ChIP_R, GCTCGGCATTCGTGTGTACC; SFRP1_ChIP_F, ACGCCGTGATCCATTCCC and SFRP1_ChIP_R, CGGCTCAACACCCCTTAAAAA; and ACTB_ChIP_F, GCCAACGCCAAAACTCTCC and ACTB_ChIP_R, CAGTGCAGCATTTTTTTACCCC.

### Cell viability assay

To assess cell proliferation, a 3-(4,5-dimethyl-2-thiazolyl)-2,5-diphenyl-2*H*-tetrazolium bromide (MTT)-based protocol was used. Directly after electroporation, cells were seeded at a density of 2500 (HUH6 and HepT1) or 5000 (HepG2) cells per well in a 96-well format (Nunc, Wiesbaden, Germany) in 100 μL RPMI medium. Ten microliters of MTT labeling agent (5 mg/mL, Sigma) was added to each well and incubated at 37 °C for 4 h. One hundred microliters of a SDS-HCl solution was added to each well and mixed thoroughly. Microplate was incubated overnight at 37 °C in a humidified chamber. The absorbance of this colorimetric reaction was quantified on the GENios reader (Tecan, Männedorf, Switzerland) by measuring at a wavelength of 595 nm.

### Cell migration assay

Transfected HB cells were seeded into 12-well plates and grown to complete confluency. A wound of approximately 1 mm was inflicted to cell monolayers with a pipette tip. The wells were washed with PBS to remove detached cells and incubated at 37 °C for a maximum time of 120 h. Images were taken every 24 h after scratching, and the wound widths were measured with ImageJ (Rasband, W.S., ImageJ, US National Institutes of Health, Bethesda, MD, USA).

### RNA sequencing

Total RNA of siUHRF1 and control transfected HUH6 cells was quality checked and quantitatively measured using the RNA 6000 nanokit on a 2100 Bioanalyzer (Agilent Technologies, Santa Clara, CA, USA). Coding transcriptomes were enriched from 1 μg total RNA in solution with TruSeq non-stranded RNA v2 kits (Illumina, San Diego, CA, USA) and sequenced as 100 bp paired-end runs on a HiSeq2500 system (Illumina) generating 35–79 million mapped reads. The STAR aligner v 2.4.2a [23] with modified parameter settings (--twopassMode=Basic) is used for split-read alignment against the human genome assembly hg19 (GRCh37) and UCSC knownGene annotation. To quantify the number of reads mapping to annotated genes, we use HTseq-count v0.6.0 [24]. The R Bioconductor package DESeq2 [25] with modified standard settings (no fold-change shrinkage) is used to investigate differences in gene expression.

### Gene set enrichment analysis

Enrichment analysis of gene ontology and hallmark gene sets was performed by using the top 1000 differentially expressed genes of the RNA sequencing results as input for the latest version of the Molecular Signatures Database (MSigDB) developed by the Broad Institute [26]. Gene sets were selected by statistical significance (*p* < 0.05) and the calculated representation factor. The representation factor is the number of overlapping genes divided by the expected number of overlapping genes drawn from two independent groups. A representation factor > 1 indicates more overlap than expected of two independent groups, and a representation factor < 1 indicates less overlap than expected.

### Statistical analysis

Data were expressed as mean ± standard deviation or ±standard error of the mean and statistically subjected to Student’s unpaired *t* test. Kaplan-Meier estimates of specific survival time in the various groups were compared using the log-rank Mantel-Cox test. Gene expression correlation was performed with GraphPad Prism version 6.0 using Spearman’s rank correlation. A level of *p* < 0.05 was considered to be significant.

## Results

### UHRF1 repression complex binds to HB-specific TSG promoter regions

**Fig. 2:**
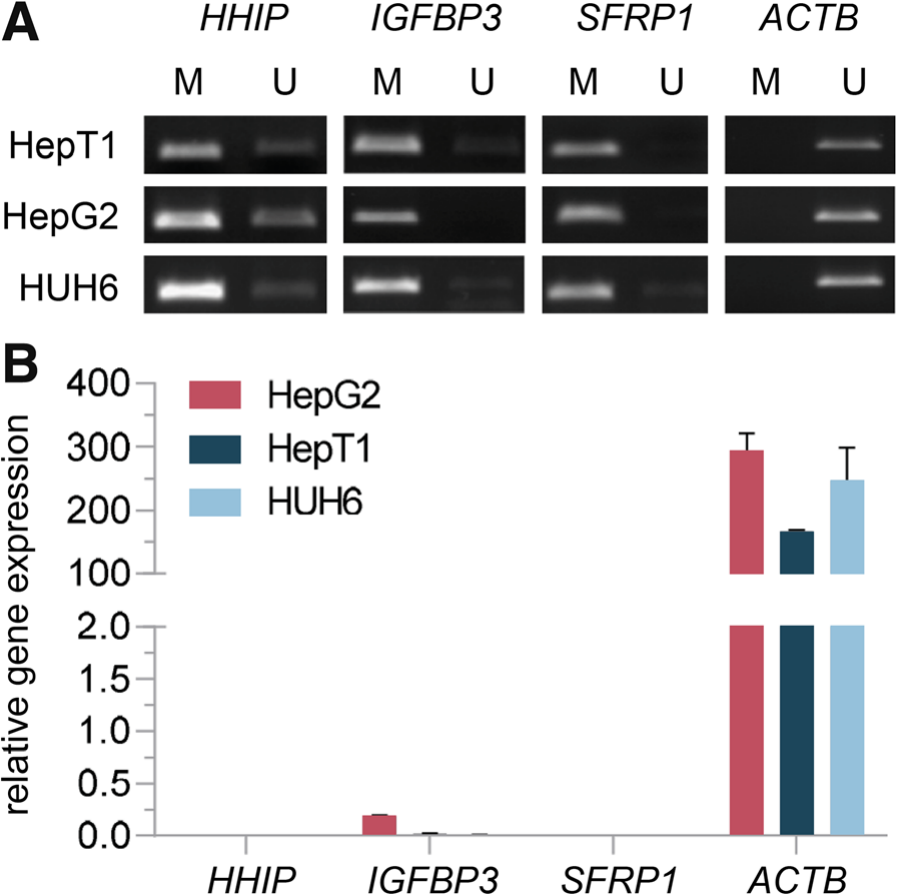
**a** Methylation status (U, unmethylated; M, methylated) of the *HHIP*, *IGFBP3*, *SFRP1*, and *ACTB* promoter region was determined for the indicated cell lines by MSP. Representative images of MSP experiments are shown. **b** Relative expression levels of indicated genes compared to the *TBP* housekeeping gene in three liver tumor cell lines are given. The average of three independent experiments is shown

We used chromatin immunoprecipitations (ChIP) to identify enrichment of the UHRF1 repression complex (Fig. 1a) on chromatin of HB cells. We found all three complex partners, namely UHRF1, DNMT1, and USP7, to be enriched on the promoter regions of the HB-specific TSGs *HHIP*, *IGFBP3*, and *SFRP1* (Fig. 1b). Furthermore, ChIP analysis of the repressive H3K9me2 and activating H3K4me2 histone marks showed enrichment of H3K9me2, while H3K4me2 was not detected (Fig. 1c). The enrichment for RNA polymerase II (RNAPII) was negligible, indicating very low or no active transcription of those genes. Subsequent methylation-specific polymerase chain reaction (MSP) revealed hypermethylated promoter regions of those three genes (Fig. 2a). Quantitative mRNA analysis of *HHIP*, *IGFBP3*, and *SFRP1* confirmed the virtual silencing of gene expression (Fig. 2b). In sharp contrast, the housekeeping gene *ACTB* did not show any enrichment for UHRF1 and the repressive H3K9me2 mark, but high levels of H3K4me2 and RNAPII, which was accompanied by an unmethylated promoter and a high transcriptional activity. These data suggest that the trimeric UHRF1 repression complex might play a crucial role in deep silencing of the TSGs *HHIP*, *IGFBP3*, and *SFRP1* in HB via combined epigenetic mechanisms, comprising repressive histone modification and DNA methylation.

### *UHRF1* knockdown leads to re-expression of TSGs through epigenetic modifications

**Fig. 3:**
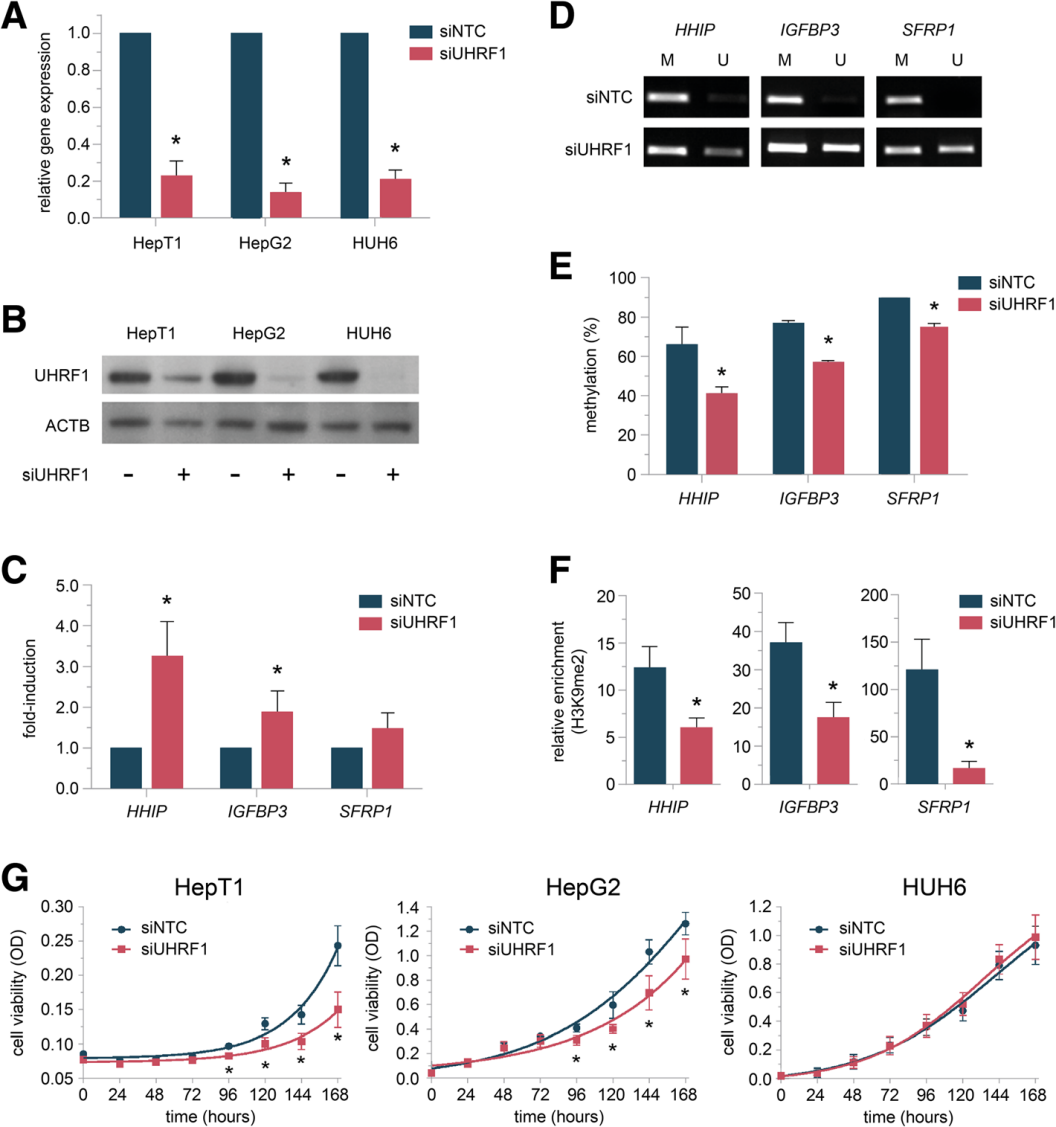
**a** Knockdown of *UHRF1* in indicated cell lines. Relative gene expression in cells transfected with siRNA against *UHRF1* compared to negative control (siNTC) after 48 h is shown. Data represents average of three independent experiments. **b** Western blot analysis of HepT1, HepG2, and HUH6 cells 48 h after *UHRF1* knockdown. **c** Relative expression levels of indicated genes in HUH6 cells 48 h after *UHRF1* knockdown compared to negative control. The average of three independent experiments is shown. **d** Methylation status (U, unmethylated; M, methylated) of the *HHIP*, *IGFBP3*, and *SFRP1* promoter region 48 h after *UHRF1* knockdown was determined by MSP in HUH6 cells. Representative images of MSP experiments are shown. **e** Pyrosequencing results of promoter regions of indicated genes in HUH6 cells 48 h after *UHRF1* knockdown of three independent experiments are shown. **f** ChIP analyses in HUH6 cells 48 h after *UHRF1* knockdown. The relative enrichment of H3K9me2 at the indicated gene promoter regions is shown. **g** Cell viability of HepG2, HepT1, and HUH6 cells as evaluated by MTT assay at the indicated time points after *UHRF1* knockdown. Values represent means ± standard deviation of three independent experiments performed in duplicates. Statistical significance of all experiments was calculated using *t* test (*p* < 0.05)

To evaluate the effect of *UHRF1* depletion in HB cells, we performed knockdown experiments, which resulted in a strong reduction of *UHRF1* both on the mRNA and the protein level (Fig. 3a, b). Depletion of *UHRF1* led to a significant re-expression of *HHIP* and *IGFBP3* (Fig. 3c). While the re-expression of *SFRP1* was not significant, we still observed an increase of *SFRP1* expression upon *UHRF1* knockdown. To further elucidate the role of the *UHRF1* repression complex, we performed MSP upon knockdown of *UHRF1*, *USP7*, and *DNMT1*, respectively. *UHRF1* knockdown led to a significant demethylation of DNA on the indicated TSG promoter regions (Fig. 3d). Quantification of those differentially methylated loci by pyrosequencing revealed a methylation decrease of 25, 20, and 15% for *HHIP*, *IGFBP3*, and *SFRP1*, respectively, upon *UHRF1* knockdown (Fig. 3e). This decrease in methylation correlates well with the strength of TSG re-expression. In addition, *UHRF1* knockdown also led to loss of the repressive H3K9me2 mark at those loci (Fig. 3f). *USP7* knockdown had no significant effect on the epigenetic marks regulating those genes and also did not result in gene reactivation (data not shown). To investigate the effect of those epigenetic changes and the re-expression of TSGs on HB cell proliferation, we performed viability assays over the course of 1 week following the knockdown. *UHRF1*-depleted HB cells showed a significant growth reduction in two out of three cell lines over time (Fig. 3g). This data suggests a combined derepressive effect of *UHRF1* knockdown on TSGs by alleviating repressive DNA and histone modifications, leading to a subsequent growth inhibition in HB cells.

### RNA sequencing reveals HB-specific transcriptional changes upon *UHRF1* knockdown

**Fig. 4:**
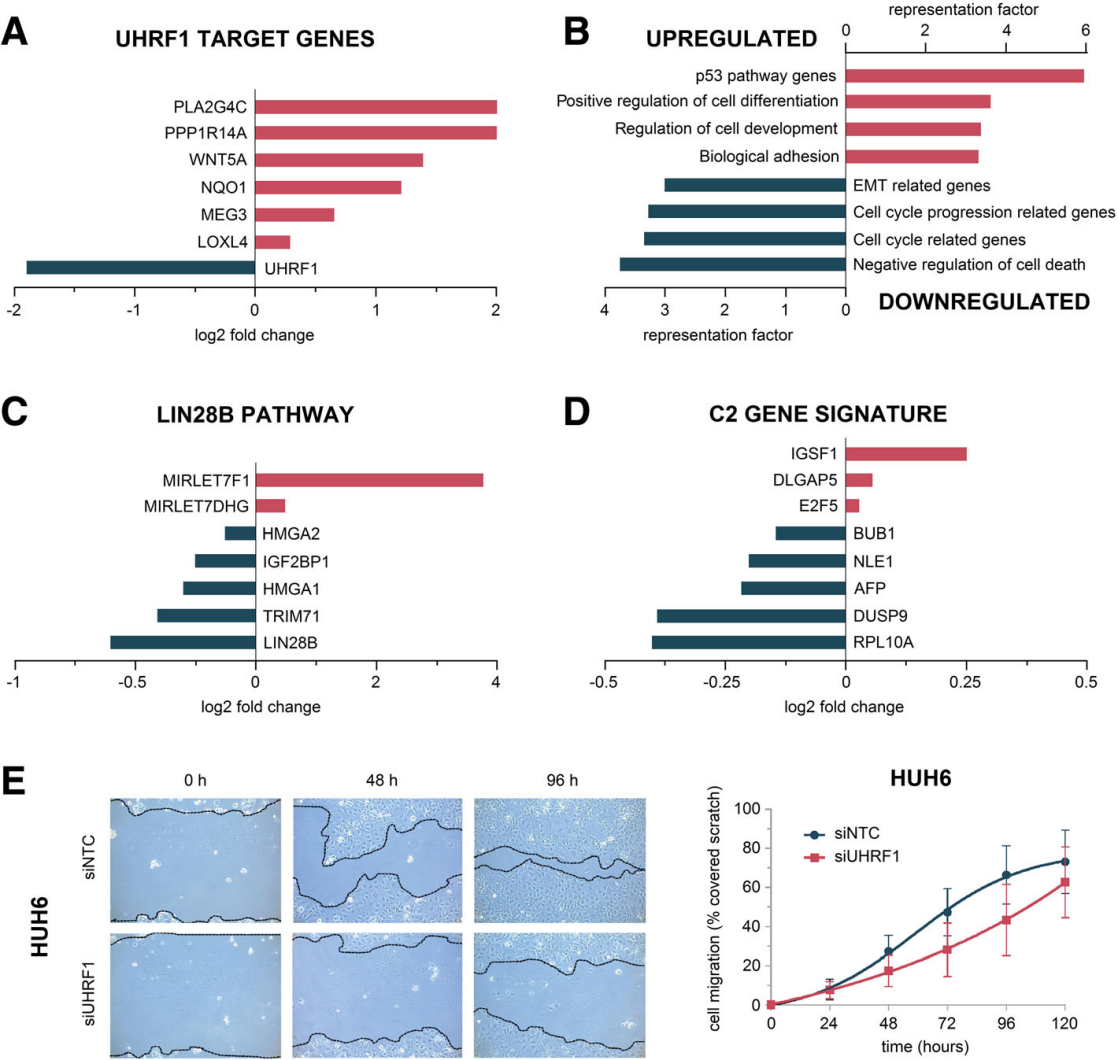
RNA sequencing results upon *UHRF1* knockdown in HUH6 cells. Log_2_ fold change of **a** known UHRF1 target genes, **c** genes related to the LIN28B pathway, and **d** genes highly expressed in the adverse C2 gene signature. **b** Significant enrichment of up- or downregulated genes within the indicated gene ontology and hallmark gene sets. Enrichment is indicated by the corresponding representation factor of each gene set. **e** Scratch assay. Representative images of HUH6 cells transfected with siRNA against *UHRF1* migrating into a cell-free space at 0, 48, and 96 h compared to negative control (siNTC). Quantitative evaluation of scratch closure shows the mean values of two independent experiments

In order to assess the effect of *UHRF1* depletion on the transcriptome, we performed RNA sequencing of HB cells upon *UHRF1* knockdown. As expected, we found *UHRF1* to be strongly downregulated and known UHRF1-repressed target genes [27–29] to be upregulated after *UHRF1* knockdown, thus emphasizing the significance of our RNA sequencing results (Fig. 4a).

To evaluate the general effect of *UHRF1* depletion in HB cells, we then used the top 1000 differentially expressed genes as input for the analysis of gene ontology and hallmark gene sets (Fig. 4b). The analysis showed significant upregulation of genes involved in the promotion of cell differentiation and development as well as cell adhesion. Genes involved in the p53 pathway were also significantly upregulated, indicating activation of this tumor suppressive pathway upon *UHRF1* knockdown. Conversely, *UHRF1* depletion led to significant downregulation of genes involved in epithelial-mesenchymal transition (EMT), cell cycle progression, and negative regulation of cell death. In order to evaluate the functional effect of downregulated EMT genes of *UHRF1*-depleted HUH6 cells, we performed cell migration assays in the identical cell line upon *UHRF1* knockdown. *UHRF1*-depleted cells showed a markedly slower cell migration compared to their control-transfected counterparts (Fig. 4e).

In recent years, LIN28B has been shown to play a pivotal role in HB initiation and progression [30]. Interestingly, we found *LIN28B* and its downstream targets to be downregulated in *UHRF1*-depleted HB cells (Fig. 4c). In agreement with this finding, we detected upregulation of *LET7* species, which are commonly suppressed by LIN28B in HB.

In HB, a prognostic 16-gene classifier has been established that discriminates between two subclasses of tumors, the so-called C1 subclass that is associated with early tumor stage and favorable patient outcome and the C2 subclass that is linked to metastases, vascular invasion, advanced tumor stage, and poor prognosis [5]. Since HB cell lines initially showed the adverse C2 expression signature, we looked for changes in the eight genes that exhibit high expression levels within the C2 signature. Notably, five of those genes were downregulated upon *UHRF1* depletion (Fig. 4d), indicating a shift towards the favorable C1 signature, which is associated with low expression levels of those genes. Real-time qPCR analysis confirmed the downregulation of C2- and LIN28B-associated genes upon *UHRF1* knockdown (Additional file 1: Figure S1). These findings suggest a more global role of *UHRF1* in HB that goes well beyond the activation of only a few TSGs.

### *UHRF1* overexpressed in high-risk HB

**Fig. 5:**
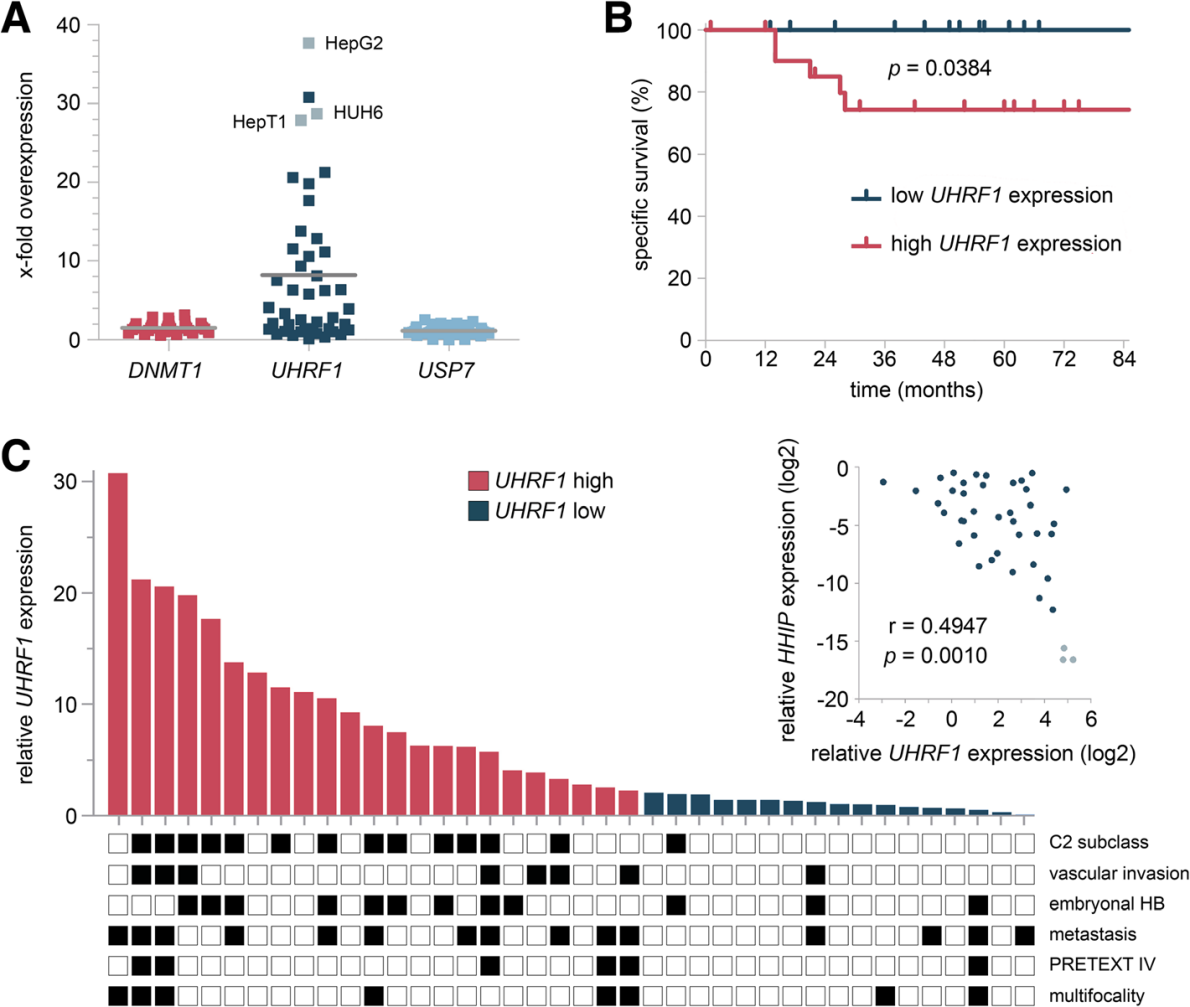
**a** The relative expression of *DNMT1*, *UHRF1*, and *USP7* in 40 primary HB and three HB cell lines (depicted in gray) compared to the mean of seven normal liver tissues are given. **b** Overall survival was calculated as time from diagnosis to death of the disease and is plotted for 40 HB patients. Statistical significance was calculated using the Mantel-Cox test. **c** Individual *UHRF1* expression levels of 40 primary HBs are shown with the occurrence of important clinicopathological characteristics depicted as black boxes below. Inset: Expression levels of *UHRF1* and *HHIP* in primary tumor tissues (dark blue dots) and tumor cell lines (light blue dots) were plotted against each other, and Spearman’s rank correlation was performed

When we investigated the expression levels of all three subunits of the trimeric repression complex, we found only *UHRF1* to be overexpressed in primary tumor samples and HB cell lines when compared to normal liver tissue (Fig. 5a). Overexpression of *UHRF1* has been reported in a variety of human malignancies and is often predictive for higher tumor stages and poor patient outcome [20]. Indeed, we found that high *UHRF1* expression levels were significantly associated with poor survival in HB patients (Fig. 5b). Of note, when comparing *UHRF1* expression levels with previously published expression levels of the tumor suppressor gene *HHIP* [21] in primary tumor tissues and the three cell lines, we also found a significant inverse correlation of both genes (Fig. 5c, inset).

Interestingly, we also found tumors exhibiting the high-risk C2 signature to be significantly associated with high expression levels of *UHRF1* (Fig. 5c). Consequently, patients with vascular tumor invasion, the unfavorable embryonal histology, metastases, large PRETEXT IV, and multifocal tumors predominantly had high expression of *UHRF1*. These data suggest that, consistent with findings in other solid tumors, *UHRF1* expression might be a prognostic marker for patients suffering from HB.

## Discussion

Genetically, HB is a very simple tumor with one of the lowest mutation rates ever reported for any malignancy [31]. Characterizing HB by its genetic events has only limited significance when it comes to risk stratification and treatment selection. In addition, genetic mutations do not explain HB tumorigenesis sufficiently and previous studies made clear that epigenetic phenomena play a key role in HB development [10, 32].

While a number of epigenetic events, such as silencing of TSGs in HB, have been described in the past, the underlying molecular mechanisms remain largely unknown. Here, we identified a trimeric repression complex that is located at HB-specific TSG sites and is capable of conveying strong transcriptional repression of those genes by combined histone and DNA modification. Knockdown of *UHRF1*, which appears to be the critical subunit of the trimeric complex, leads to histone and DNA demethylation, re-expression of TSGs, and growth inhibition in HB cells. Reduced viability upon knockdown might be a result of the downregulation of genes involved in cell cycle progression and the negative regulation of cell death. UHRF1 has been shown to be a negative regulator of the p53 tumor suppressor pathway, and growth inhibition in HB cells might be augmented by the activation of this pathway following *UHRF1* depletion [33]. Knockdown-related upregulation of genes involved in cell differentiation and development might be a combined effect of p53 activation and the re-expression of TSGs that are capable of blocking aberrant growth signaling in HB cells. Notably, *UHRF1* knockdown led to downregulation of the HB-initiating LIN28B pathway and induced a shift towards the more favorable C1 signature. It also led to the upregulation of genes involved in cell adhesion and downregulation of EMT-related genes, thus promoting a more anti-metastatic phenotype. Our results strongly indicate that *UHRF1* represents an interesting molecular target for novel treatment strategies in HB.

As epigenetic aberrations are potentially reversible, a number of epigenetic therapy approaches have been developed in the last few years, some of which have shown great promise in the treatment of cancer [34]. In fact, we have recently shown that HDAC inhibition constitutes a potential epigenetic treatment option for high-risk HB patients [8]. Targeting *UHRF1* in HB holds the potential to further improve patient outcome, while reducing doses and toxicities of untargeted chemotherapeutic agents. Notably, the first in vitro studies of newly discovered *UHRF1* inhibitors have shown encouraging results in several cancer cell lines [35–38].

Furthermore, *UHRF1* overexpression seems to be a common feature in many malignancies and has therefore been suggested as a universal biomarker for cancer [20, 39–41]. Our systematic expression analysis of a large set of primary HB uncovered *UHRF1* overexpression to be a potential high-risk feature of HB, as high *UHRF1* expression levels were significantly correlated with poor survival in HB patients. Moreover, the C2 subtype of the 16-gene signature that has been shown to predict poor prognosis in HB was also significantly associated with high *UHRF1* expression.

## Conclusions

Collectively, our findings suggest that UHRF1 is critical for aberrant epigenetic modifications and sustained growth signaling in HB. The unique capability of UHRF1 to convey repressive DNA and histone modifications highlights its potential to induce deep TSG silencing and other oncogenic events. UHRF1 therefore constitutes a promising target for novel therapeutic approaches in HB patients. Its overexpression in patients with high-risk features makes UHRF1 also a strong candidate for a prognostic biomarker in HB.

## Additional file

Additional file 1: **Figure S1.** Relative RNA expression levels of indicated genes in HUH6 cells 24 h after *UHRF1* knockdown compared to control-transfected cells. Data were normalized to the expression level of the housekeeping gene *TBP*. The average of two independent knockdown experiments is shown. Statistical significance of all experiments was calculated using *t* test (*p* < 0.05). (PNG 110 kb)

## Abbreviations

ACTB: Beta actin
ChIP: Chromatin immunoprecipitation
CpG: Cytosine-phospho-guanine
DNMT1: DNA methyltransferase 1
EMT: Epithelial-mesenchymal transition
H3K4me2: Histone 3 lysine 4 di-methylation
H3K9me2: Histone 3 lysine 9 di-methylation
HB: Hepatoblastoma
HDAC: Histone deacetylase
HHIP: Hedgehog-interacting protein
IGFBP3: Insulin-like growth factor-binding protein 3
LIN28B: Lin-28 homolog B
MSP: Methylation-specific polymerase chain reaction
p53: Tumor protein p53
RNAPII: RNA polymerase II
SFRP1: Secreted frizzled-related protein
SRA: SET- and RING-associated
TSG: Tumor suppressor gene
TTD: Tandem tudor domain
UHRF1: E3 ubiquitin-like containing PHD and RING finger domain 1
USP7: Ubiquitin-specific-processing protease 7
