# Supplementary figures and images for: Overexpression of UHRF1 promotes silencing of tumor suppressor genes and predicts outcome in hepatoblastoma
Source: Clin Epigenetics. 2018 Mar 2;10:27. doi: 10.1186/s13148-018-0462-7 (PMC5833129; doi:10.1186/s13148-018-0462-7)

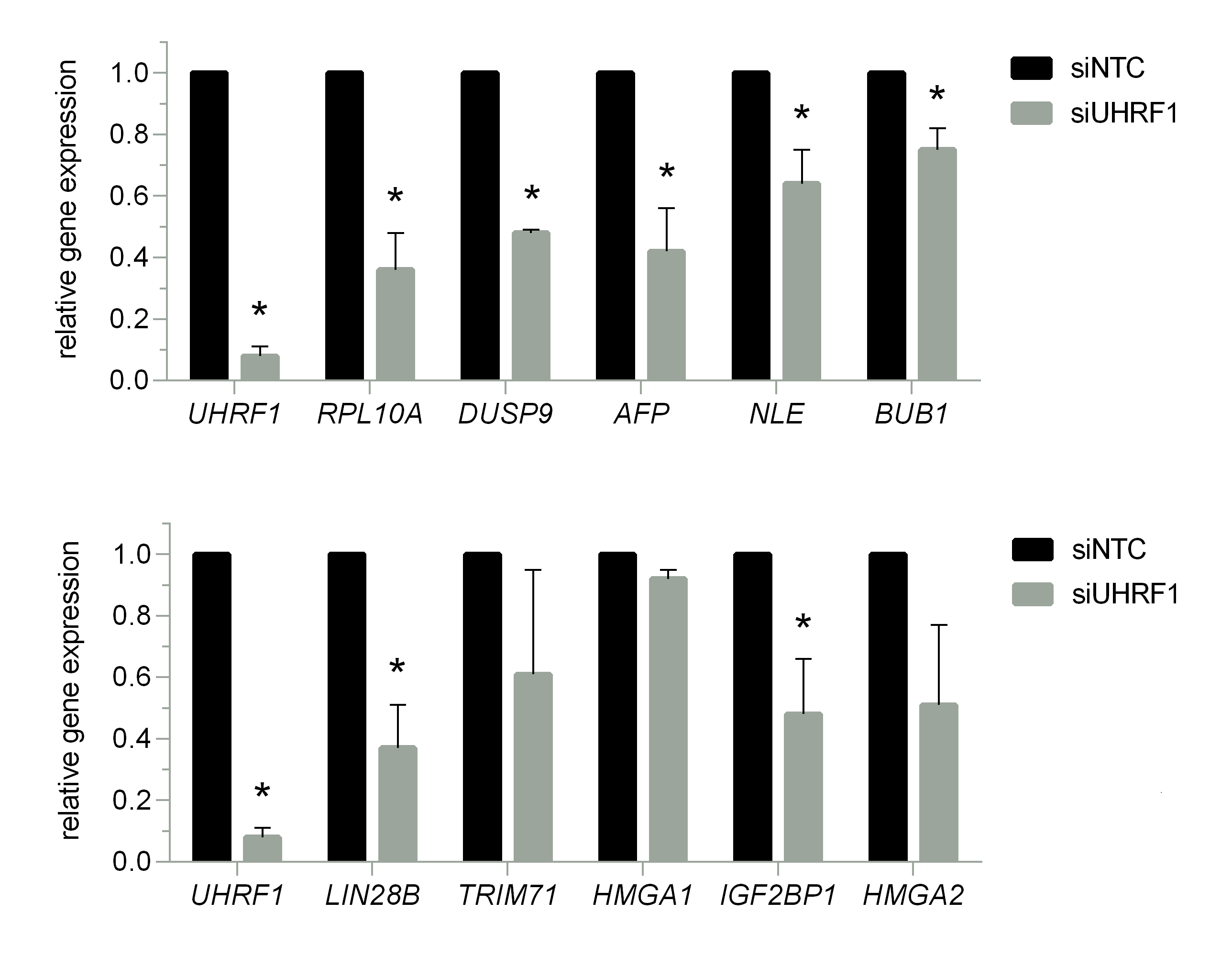

Supplement: Supplementary file 1 — Figure S1. Relative RNA expression levels of indicated genes in HUH6 cells 24 h after UHRF1 knockdown compared to control-transfected cells. Data were normalized to the expression level of the housekeeping gene TBP. The average of two independent knockdown experiments is shown. Statistical significance of all experiments was calculated using t test (p < 0.05). (PNG 110 kb) [file 13148_2018_462_MOESM1_ESM.png]
